# Integrated network pharmacology and serum metabolomics approach deciphers the anti-colon cancer mechanisms of Huangqi Guizhi Wuwu Decoction

**DOI:** 10.3389/fphar.2022.1043252

**Published:** 2022-10-13

**Authors:** Boyu Pan, Yafei Xia, Senbiao Fang, Jun Ai, Kunpeng Wang, Jian Zhang, Chunshuang Du, Yuzhou Chen, Liren Liu, Shu Yan

**Affiliations:** ^1^ Tianjin Key Laboratory of Acute Abdomen Disease Associated Organ Injury and ITCWM Repair, ITCWM Hospital, Tianjin University, Tianjin, China; ^2^ Department of Molecular Pharmacology, Tianjin Medical University Cancer Institute and Hospital, National Clinical Research Center for Cancer, Key Laboratory of Cancer Prevention and Therapy, Tianjin’s Clinical Research Center for Cancer, Tianjin, China; ^3^ Department of Laboratory Animal Science, Tianjin Medical University, Tianjin, China; ^4^ Department of Oral and Maxillofacial Surgery, Stomatological Hospital, Tianjin Medical University, Tianjin, China; ^5^ Department of Pharmacy, Tianjin Medical University Cancer Institute and Hospital, National Clinical Research Center for Cancer, Key Laboratory of Cancer Prevention and Therapy, Tianjin’s Clinical Research Center for Cancer, Tianjin, China; ^6^ Department of Pharmaceutics, College of Traditional Chinese Medicine, Tianjin University of Traditional Chinese Medicine, Tianjin, China

**Keywords:** Huangqi Guizhi Wuwu Decoction (HGWD), colon cancer (CC), network pharmacology, serum metabolomics, CYP2E1, new use of old medicine

## Abstract

Huangqi Guizhi Wuwu Decoction (HGWD), as a classic Chinese herbal decoction, has been widely used in treating various diseases for hundreds of years. However, systematically elucidating its mechanisms of action remains a great challenge to the field. In this study, taking advantage of the network pharmacology approach, we discovered a potential new use of HGWD for patients with colon cancer (CC). Our *in vivo* result showed that orally administered HGWD markedly inhibited the growth of CC xenografts in mice. The subsequent enrichment analyses for the core therapeutic targets revealed that HGWD could affect multiple biological processes involving CC growth, such as metabolic reprogramming, apoptosis and immune regulation, through inhibiting multiple cell survival-related signalings, including MAPK and PI3K-AKT pathways. Notably, these *in silico* analysis results were most experimentally verified by a series of *in vitro* assays. Furthermore, our results based on serum metabolomics showed that the lipid metabolic pathways, including fatty acid biosynthesis and cholesterol metabolism, play key roles in delivery of the anti-CC effect of HGWD on tumor-bearing mice, and that cytochrome P450 family 2 subfamily E member 1 (CYP2E1) is a potential therapeutic target. Together, our integrated approach reveals a therapeutic effect of HGWD on CC, providing a valuable insight into developing strategies to predict and interpret the mechanisms of action for Chinese herbal decoctions.

## Introduction

Colon cancer (CC) is a prevalent carcinoma of the digestive system (Brody, 2015). The incidence of CC and its associated mortality rate are increasing worldwide (Tariq and Ghias, 2016; Petrelli et al., 2020). At present, surgical resection, chemotherapy and targeted therapy are the main treatment options for CC (Sanoff et al., 2008). Although some important advances have been made in CC treatment, the 5-year survival rate is low (Center et al., 2009; Edwards et al., 2010). Therefore, development of new strategies is urgently needed to enhance the survival as well as prognostic outcomes of patients with CC.

**FIGURE 1:**
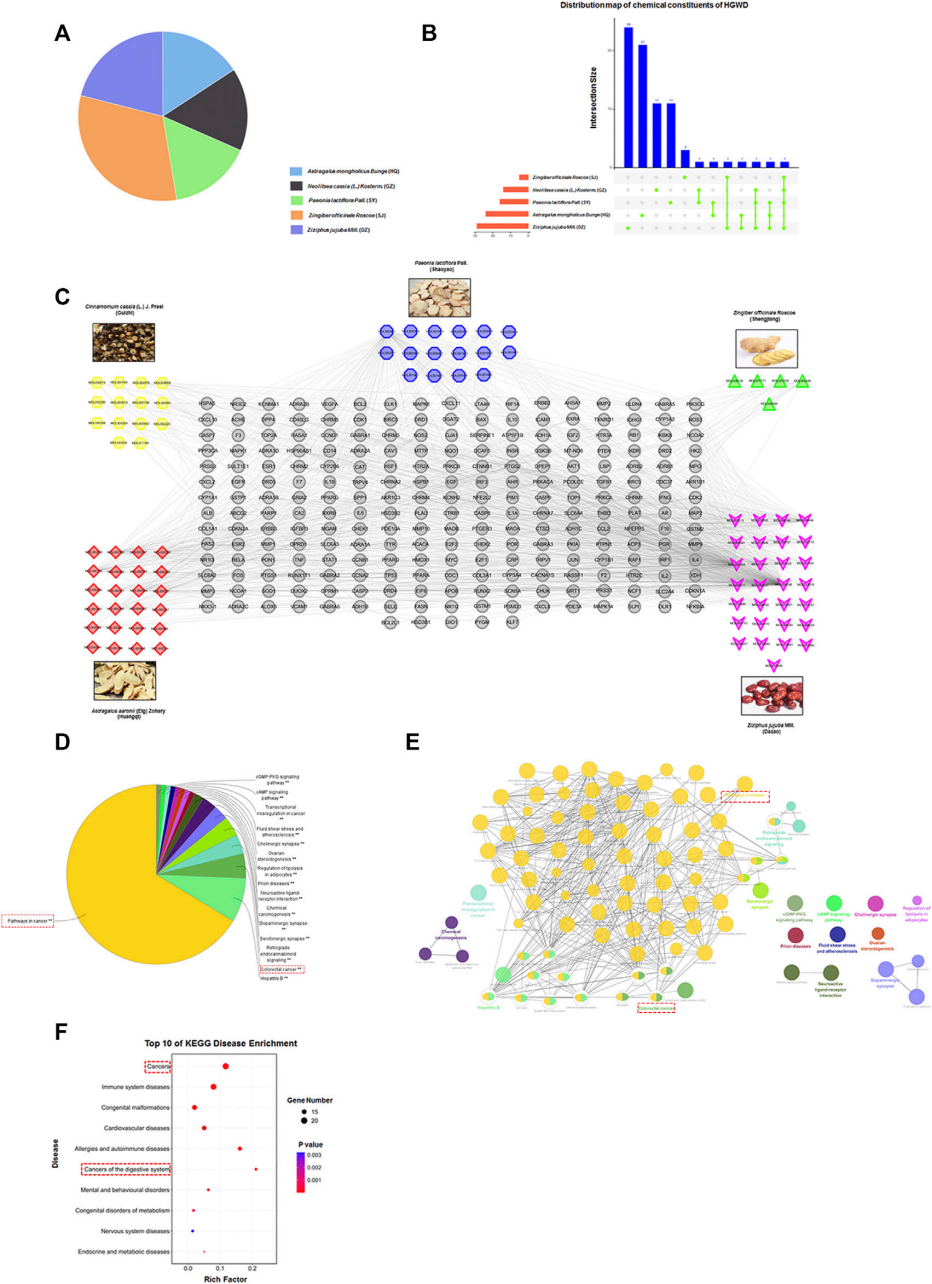
Establishment of HGWD candidate ingredient-target systematic network and enrichment analyses. **(A)** The quality matching of five ingredients from HGWD (HQ, SY, GZ, SJ and DZ). **(B)** The distribution map of chemical constituents of HGWD. **(C)** The systematic network was established by linking potential active ingredients and their putative drug targets in HGWD. **(D–F)** Putative drug targets were respectively enriched in pathway as well as disease using ClueGO and KOBAS 3.0, (*p* < 0.05).

As traditional Chinese medicine (TCM) components, Chinese herbal medicines (CHM) have been extensively used as therapeutic options for various difficult miscellaneous diseases in China, such as cancers (Tang et al., 2008; Chen et al., 2018a). Huangqi Guizhi Wuwu Decoction (HGWD) is a TCM prescription from Han Dynasty, *Synopsis of Golden Chamber*. It was lately listed among the first 100 ancient classic prescriptions in China. HGWD is made up of five CHMs: *Astragalus mongholicus Bunge* (Chinese name: Huangqi, HQ), Neolitsea cassia (L.) Kosterm (Chinese name: Guizhi, GZ), Paeonia lactiflora Pall. (Chinese name: Shaoyao, SY), Zingiber officinale Roscoe (Chinese name: Shengjiang, SJ), and Ziziphus jujuba Mill (Chinese name: Dazao, DZ) (Figure 1A). Above all five species were fully validated using a “Medicinal Plant Names Services” key search tool (http://mpns.kew.org/mpns-portal/?_ga=1.111763972.1427522246.1459077346). HGWD is traditionally mainly used to treat syndromes that include blood arthralgia, skin numbness, slight astringency and tense pulse (Liu et al., 2021). HGWD has good pharmacological effects in cases of diabetic peripheral neuropathy (Zheng et al., 2019), cardiovascular and cerebrovascular diseases (Zheng et al., 2020), periarthritis of the shoulder (Zhang et al., 2020), cervical spondylosis (Liang et al., 2020), rheumatoid arthritis (Wang et al., 2020a), and oxaliplatin-induced peripheral neurotoxicity (Wei et al., 2020). However, the underlying mechanisms of HGWD in treatment of other diseases remain to be systematically studied.

Recently, metabolomics evaluates changes in the metabolic profiles under various physiological and pathological states by comparing the differences among metabolites, thereby further revealing the action mechanisms of drugs (Guijas et al., 2007). TCM has emphasized the overall concept and syndrome differentiation and treatment. It infers the evolution of the overall state of patients based on subtle changes in their syndromes. Therefore, TCM shares similarities with metabolomics research. Due to its complexity, TCM often exhibits the characteristics of multi-target and mutual synergy when used to treat various clinical diseases. Thus, metabolomics analysis objectively reflects the metabolic changes that occur after drug administration and the metabolic profiles of biomarkers at different stages, which can provide a basis for elucidation of action mechanisms of TCM.

We first performed a systematic virtual research of the action pharmacological mechanism of HGWD. We discovered the potential anti-CC effect of HGWD, a finding that suggests a potential new application of HGWD in cancer treatment. Subsequently, *in vivo* as well as *in vitro* assays were conducted under the guidance of network pharmacology (Hopkins, 2007; Hopkins, 2008; Pan et al., 2018; Pan et al., 2020; Pan et al., 2021). It was found that HGWD directly inhibits CC cell growth by simultaneously blocking multiple signaling pathways. The subsequent metabolomic analysis found that HGWD regulates important tumor-related metabolic pathways in CC-bearing mice, thus playing a key anti-CC role. Our research combined network pharmacology with metabolomics may provide a new idea for exploration of new clinical uses of classic TCM prescriptions.

## 2 Materials and methods

### Cell cultures and related reagents

Human CC cell lines (SW-480, HT-29 and HCT-15) and a murine CC cell line (MC-38) were acquired from China Infrastructure of Cell Line Resources (China) and respectively grown in DMEM and RPMI-1640 media with 10% (v/v) foetal bovine serum (FBS) and 100 U/ml penicillin/streptomycin under standard conditions (37°C and 5% CO_2_). Cell Counting Kit-8 (CCK-8) assay kits were acquired from Dojindo (Japan). Hoechst 33342 and JC-1 reagents were procured from Beyotime (China). The Annexin-V FITC apoptosis kits (#556547) were bought from BD Biosciences Pharmingen (United States). Antibodies against Caspase 9/p35/p10, Bcl-2, Bax, PARP1 and beta-actin were procured from Proteintech Group Inc. Antibodies against p-Akt (T308), p-Akt (S473) and p-ERK (T202/Y204) were bought from Cell Signalling Technology (CST), and antibodies against CYP2E1 were acquired from Sino Biological Inc. The five CHMs used to prepare HGWD were acquired from Hebei Kangbo Pharmaceutical Co., Ltd (China). The batch numbers of the five herbs were as follows: HQ (No. 212331121), SY (No. 210630703), GZ (No. 212121101), SJ (No. 210370721), and DZ (No. 216330301). All the above medicinal materials were stored in a dry and dark environment. The species identification of the five CHMs was confirmed by Professor Lijuan Zhang (Department of Traditional Chinese Medicine Identification, College of Traditional Chinese Medicine, Tianjin University of Traditional Chinese Medicine). HGWD was provided by the College of Traditional Chinese Medicine, Tianjin University of Traditional Chinese Medicine. It was decocted in accord with quality control standard of the *Chinese Pharmacopoeia*. Mass (g) ratio of the five CHMs in HGWD were: HQ:GZ:SY:SJ:DZ = 3:3:3:6:4. The concentration of the stock decoction was 0.05 g/ml. In subsequent experiments, the 0.05 g/ml stock was filter-sterilized using a filter membrane (0.22 μm) and diluted to appropriate doses. The stock solution described above was maintained at −80°C for posterity.

### Screening of potential active ingredients in HGWD

The traditional Chinese medicine systems pharmacology database and analysis platform (TCMSP, https://www.tcmsp-e.com/) is a database and visualization platform that was developed based on systems pharmacology framework for traditional CHMs (Ru et al., 2014). In the present study, the major active ingredients in HGWD were retrieved *via* the TCMSP online platform using the five CHMs as keywords. All of the ingredients identified were screened for activities based on their absorptions, metabolism, distributions, and elimination (ADME)-associated parameters. Parameters were set as: drug-like properties (DL) ≥ 0.18 and oral bioavailability (OB) ≥ 30%. Meanwhile, we searched the China National Knowledge Infrastructure (CNKI) literature library (www.CNKI.net) to identify the major active ingredients of each of the five CHMs that did not fully meet the above mentioned parameters.

### Assessment of drug targets for HGWD and disease targets associated with CC

Protein targets corresponding to the obtained active ingredients were further retrieved using the TCMSP database. The gene name corresponding to every protein target was retrieved *via* the Uniprot online database (http://www.uniprot.org/). Datasets were then constructed after the species origin was verified as “Human”. In addition, known disease targets that were closely associated with CC were acquired from the Gene Expression Omnibus (GEO) database (https://www.ncbi.nlm.nih.gov/geo/), and the data obtained in this manner included three gene expression microarray datasets (GSE13471, GSE44076, and GSE33113) from human CC as well as paracancerous tissues. Protein-protein interactions (PPI) between drug and disease targets were analysed using BisoGenet, a core plugin of the Cytoscape software (Version 3.2.1). Final findings were incorporated into a single-result diagram that included six independent analyses of PPI data.

### Establishment of the systematic network and correlation enrichment analyses

First we established the PPI networks of the potential targets of HGWD and the CC-associated disease targets, and performed image visualization using Cytoscape software (Shannon et al., 2003). The two PPI networks were then merged, after which topological parameters for every node in the merged network were determined by CytoNCA, an important plugin provided with the Cytoscape software. We considered the nodes in the merged network system that had at least twice the median of degree centrality (DC) vital nodes and generated a new relationship network containing these nodes. To screen for the possible core targets of HGWD in CC treatment, we mined the nodes in the new network that had topological parameters higher than medians of DC, closeness centrality (CC’) and betweenness centrality (BC). These targets were the most significant among all nodes. The obtained potential core targets were then subjected to biological function and signaling pathway enrichment analysis using FUNRICH (version 3) software and the Database for Annotation, Visualization and Integrated Discovery (DAVID) v6.8 (https://david.ncifcrf.gov/), respectively.

### Mouse xenograft assays

Male C57BL/6 mice (aged 4 weeks) were acquired from Beijing Vital River Laboratory Animal Technology Co., Ltd (China). Experimental assays were conducted in accord with recommendations of the Ethical Committee of the Integrated Traditional Chinese and Western Medicine (ITCWM) Hospital, Tianjin University. Eighteen mice were randomized into three groups (n = 6). When mice were aged 5, MC-38 cells (1.5 × 10^6^/100 μl) were inoculated subcutaneously into their right hips using a 1-ml syringe. When cancers became visible to the naked eye (10 days later), the mice were given 200 µl normal saline (blank control group) or 200 µl of HGWD solution (300 mg/kg body weight) by gavage (p.o.). The drug was administered twice daily (in the morning and in the afternoon) during the experimental period. The mice in the positive drug control groups received intraperitoneal administration of oxaliplatin (L-OHP, 4 mg/kg body weight) and 5-fluorouracil (5-FU, 20 mg/kg body weight) once every 3 days during the experimental period (Pan et al., 2018). Tumor volume was measured once daily; it was determined as: long diameter × (short diameter)^2^/2. Mice body weights were also evaluated daily. On the 14th day, the experiment was terminated, and the mice were sacrificed. Tumor specimens were photographed and weighed. During the experiment, none of the mice died. Flow cytometry was also performed to examine the numbers of CD3^+^CD4^+^ as well as CD3^+^CD8^+^ T cells infiltrating the tumors. The procedures of immunohistochemical examination have been described previously (Pan et al., 2021).

### Verification of cellular functions and molecular pathomechanisms

The processes used to assess cell viabilities and colony formation abilities were as previously reported (Pan et al., 2021). Cumulative distance of CC cell migration was determined *via* Operetta CLS high-content analysis system equipped with the Harmony software (PerkinElmer, Waltham, MA, United States). Measurements were completed in the digital phase contrast mode at 37°C and 5% CO_2_ using a ×20 long-distance objective. Moreover, EdU cell proliferation assays, apoptosis assays, mitochondrial membrane potential (MMP) assays, and real-time fluorescence-based quantitative polymerase chain reaction (qRT-PCR) as well as western blot (WB) analyses were conducted in accordance with the instructions provided with the kits and reagents. The CYP2E1 primers used in qRT-PCR verification were as follows: forward primer: GGG​AAA​CAG​GGC​AAT​GAG​AG and reverse primer: GGA​AGG​TGG​GGT​CGA​AAG​G. And the GAPDH primers used in qRT-PCR verification were also as follows: forward primer: TGC​ACC​ACC​AAC​TGC​TTA​GC and reverse primer: GGC​ATG​GAC​TGT​GGT​CAT​GAG.

### Metabolite extractions and LC-MS analysis

To extract metabolites from mouse plasma samples, 400 μl of cold extraction solvent methanol/acetonitrile/H_2_O (2:2:1, v/v/v) was supplemented to 100 mg of the sample, and thereafter vortexed. After vortexing, incubation of samples on ice was done for 20 min, after which centrifugation was done for 20 min at 14,000 g and 4°C. The supernatants were obtained and dried in a vacuum centrifuge at 4°C. For LC-MS analyses, samples were re-dissolved in 100 μl acetonitrile/water (1:1, v/v) solvent and transferred to LC vials. For untargeted polar metabolite metabolomics, extracts were assessed by a quadrupole time-of-flight mass spectrometer (Sciex TripleTOF 6,600) coupled to hydrophilic interaction chromatography *via* electrospray ionization in Shanghai Applied Protein Technology Co., Ltd. Mass spectrometry was performed in both negative ion and positive ionizations modes.

### Data processing and bioinformatics analysis

Raw MS data (wiff.scan files) were transformed to MzXML files *via* ProteoWizard MS Convert and imported into the XCMS software. Metabolites identification by MS/MS spectra with an in-house database build with authentic standards. After normalization to total peak intensity, processed data were uploaded into prior to importation into SIMCA-P (version 14.1, Umetrics, Umea, Sweden). Seven-fold cross-validations and response permutation assessments were used to investigate the models’ robustness. Variable importance in the projection (VIP) value for every variable in the OPLS-DA model was evaluated to establish its significance in the classification. Significance was assessed by unpaired Student’s *t*-test. VIP >1 and *p* < 0.05 were markedly significant.

For KEGG pathway annotations, metabolites were blasted against the online KEGG database to establish their COs and were then mapped to KEGG11 pathways. With regards to Fisher’ exact test, KEGG analyses were performed, given the whole metabolites for every pathway as the background dataset. Pathways with *p* < 0.05 were denoted as markedly altered. For hierarchical clustering, Cluster 3.0 (http://bonsai.hgc.jp/~mdehoon/software/cluster/software.htm) and Java Treeview software (http://jtreeview.sourceforge.net) were used.

### Metabolic pathway analysis and potential key target screening

The potential biological roles of related differential metabolites were evaluated by the MetaboAnalyst enrichment analysis database (http://www.metaboanalyst.ca/). MetScape, the metabolic network analysis and visualization software (http://metscape.ncibi.org./), was employed in generation of the systematic network associated with each of the metabolites and related targets. Meanwhile, we also established the PPI network by using above targets, and excavated the key target by MCODE plugin of Cytoscape and UALCAN online database (http://ualcan.path.uab.edu/) analyses.

### Molecular docking analysis

AutoDock software (4.2 versions) was used to dock the structures of three in-blood ingredients, including cinnamaldehyde, isorhamnetin and quercetin from HQ and GZ of HGWD (45 mg/kg body weight by gavage) detected by UPLC-QTOF-MS method. Then, above these ingredients were further docked with CYP2E1 (PDB code: 3E4E), respectively. The procedures of docking have been described previously (Pan et al., 2021).

### Statistical analyses

The experimental data were analyzed by the Graphpad Prism 6.0 software (United States). Data are shown as mean ± SD. The Student’s *t*-test was used to evaluate between-group differences. *p* < 0.05 denoted significance.

## Results

### Candidate active constituents and potential drug targets of HGWD

To systematically assess the pathomechanisms of HGWD, we set the ADME-related parameters using the TCMSP online database to OB ≥ 30% and DL ≥ 0.18. For the five CHMs contained in HGWD, 63 potentially active components were identified. Although some ingredients did not meet all the requirements of the above parameter settings, they were the known main components of the five CHMs and were thus included in the subsequent investigation. Another 14 main components were obtained through literature mining. Finally, the results were combined, and the duplicates were removed, leaving a total of 77 candidate active components (Figure 1B and Supplementary Table S1).

We further explored the potential drug targets of the 77 candidate components using the TCMSP database. This resulted in the acquisition of a total of 243 targets (Supplementary Table S1). Intriguingly, the candidate components had many overlapping targets, indicating that these ingredients might play key synergistic roles. Next, we constructed the drug and target network using the Cytoscape software, thereby visualizing the interaction between the systems (Figure 1C).

Next, using the ClueGO plugin in Cytoscape software, we performed KEGG pathway enrichment of the 243 potential drug targets. We found that “Pathways in cancer” ranked first and was closely related to “Colorectal cancer” (Figures 1D,E). We also conducted disease type enrichment analysis of the above drug targets and found that these targets were closely related to “Cancers” and “Cancers of the digestive system” (Figure 1F). Thus, these findings imply that HGWD has a potential therapeutic effect on CC.

### Oral administration of HGWD significantly inhibits CC cell growth in C57BL/6 mice

**FIGURE 2:**
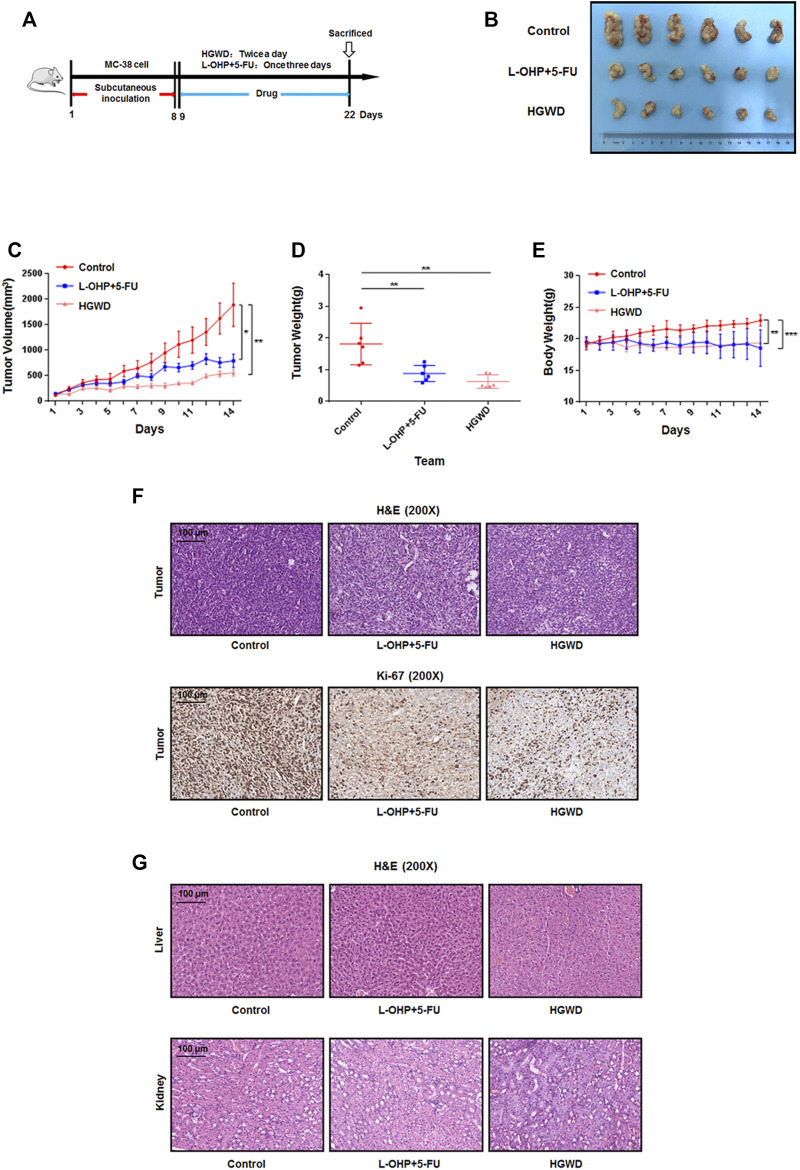
HGWD inhibited the growth of xenografted CC tumors in C57BL/6 mice **(A)** Workflow for this *in vivo* analysis. **(B)** Image of tumor sizes on day 14. **(C)** Tumor volumes were evaluated once daily for 14 successive days. **(D)** Tumors were resected on day 14 and their weights determined. **(E)** Body weights were determined once daily for 14 successive days. **(F)** H&E and immunohistochemistry staining for Ki-67 were done using tumor slides from different groups. **(G)** H&E were performed by using the liver and kidney slides from different groups. *p < 0.05, **p < 0.01 and ***p < 0.001.

To evaluate the anti-CC effects of HGWD from the perspective of *in vivo* efficacy, we treated C57BL/6 mice carrying CC xenograft tumors *via* intragastric administration of HGWD. Mice that received normal saline and a combination of clinical first-line chemotherapy drugs (L-OHP + 5-FU) served as the blank control group and the positive drug control group, respectively (Figure 2A). Compared to the blank controls, orally administered HGWD markedly suppressed MC-38 xenograft tumor growth in mice (Figure 2B). On the 14th day, the mean tumor volume in the blank control group was about 3.4 times that of HGWD treatment group (*p* < 0.01) (Figure 2C). A marked difference in tumor weights between the above two groups was consistently observed (*p* < 0.01) (Figure 2D). We also compared the efficacy of HGWD with that of the chemotherapy regimen (L-OHP+5-FU) in treatment of CC. The results showed that the CC xenograft tumors in the HGWD treatment group grew slightly more slowly than those in the L-OHP+5-FU treatment group (Figure 2B). On the 14th day, the average tumor volume and weight were also slightly low in the HGWD treatment group, relative to the L-OHP+5-FU treatment group (Figures 2C,D). Meanwhile, differences in body weights between the HGWD and L-OHP+5-FU treatment groups were not marked (Figure 2E). Haematoxylin and eosin (H&E) staining revealed that, compared to the blank controls, significant numbers of necrotic cells were presented in tumor tissues treated with HGWD or chemotherapeutic drugs. The tumor tissues also showed weaker nuclear staining after treatment with HGWD or chemotherapeutic drugs. Immunohistochemical analyses revealed that the number of Ki-67-positive cells was markedly lower in tumors treated with HGWD or chemotherapeutic drugs, relative to the blank control group, implying that HGWD had antiproliferative effects (Figure 2F). Meanwhile, treatment with HGWD or chemotherapeutic drugs had no significant toxic effects on the animals’ livers or kidneys (Figure 2G). Thus, HGWD has a direct antigrowth effect on CC *in vivo*.

### HGWD markedly inhibits the growth and decreased the CC cell motility and viability

**FIGURE 3:**
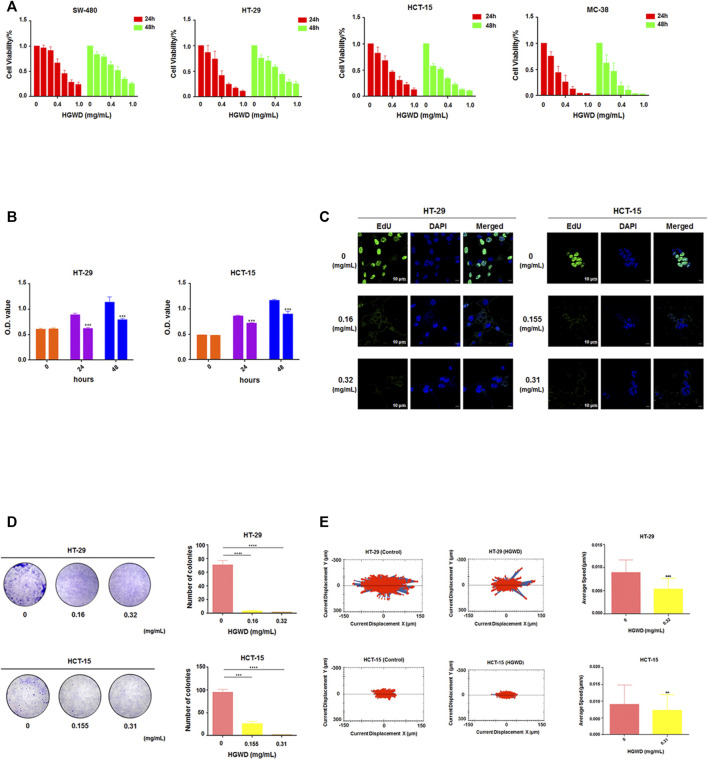
HGWD suppressed CC cell proliferation and their viability. **(A)** CCK-8 assay was done to assess CC cells viability at 24 and 48 h after HGWD treatment at various doses (0, 0.1, 0.2, 0.4, 0.6, 0.8, 1.0 mg/ml). **(B)** CCK-8 assay was done to evaluate CC cells growth ability at 24 and 48 h after HGWD treatment at 24 h IC_50_ (0.32 mg/ml and 0.31 mg/ml) in HT-29 and HCT-15 cells, respectively. ***p < 0.001. **(C)** EdU assay was conducted to assess CC cells proliferation ability after HGWD treatment for 24 h. **(D)** Cell colony formation assays were done to evaluate CC cells clonality after 24 h of HGWD treatment. ***p < 0.001 and ****p < 0.0001. **(E)** The Operetta CLS High Content Analysis System was used to assess the accumulated distance of CC cells at 24 h after HGWD treatment. **p < 0.01 and ***p < 0.001.

We conducted *in vitro* pharmacodynamic assays on HGWD using CC cells. It was established that HGWD time- and dose-dependently decreased the viability of human CC cells (SW-480, HT-29 and HCT-15) and mouse CC cells (MC-38) (Figure 3A). Respectively, the 24 h IC_50_ values for HGWD in HT-29 and HCT-15 cells (HGWD had a more marked inhibition effect on these double cell lines) were 0.327 ± 0.087 mg/ml and 0.316 ± 0.029 mg/ml. Subsequent findings showed that relative to the control group, exposures of HT-29 and HCT-15 cells to HGWD at a dose corresponding to the 24 h IC_50_ significantly inhibited the ability of cell growth (Figure 3B). In addition, cell proliferation was significantly inhibited as the dose of HGWD increased (Figure 3C). The colony formation assay revealed that clonality of 2 cells was dose-dependently decreased (Figure 3D). In addition, we observed the average cumulative distance of cell migration was smaller in HGWD treatment group than control group (Figure 3E). These findings imply that HGWD significantly inhibits the growth and viability of CC cells.

### Establishment of the PPI systematic network of HGWD anti-CC and core targets enrichment analyses

**FIGURE 4:**
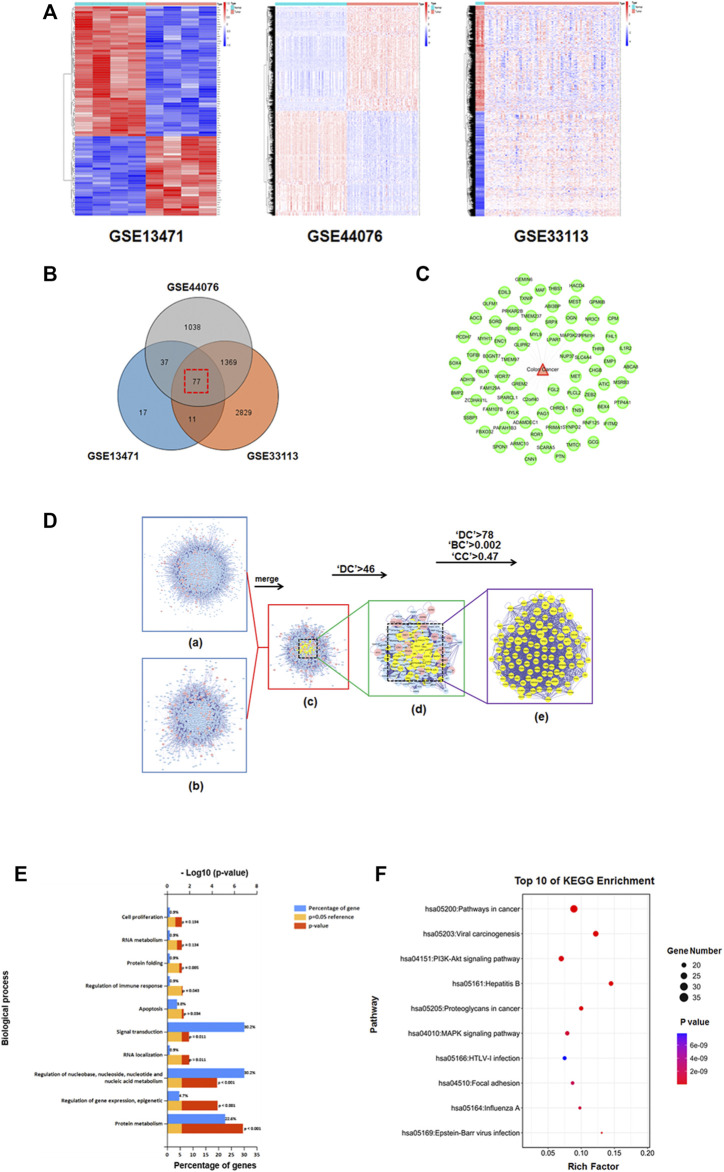
Known CC-associated targets were screened from the GEO database, and screeening the candidate core targets for HGWD against CC and enrichment analyses. **(A)** Three heat maps from GEO chips, including GSE13471, GSE44076 and GSE33113. **(B)** Venn diagram of 77 common CC-associated targets from three GEO chips. **(C)** Establishment of the CC-associated targets network. **(D)** (i) PPI network of HGWD putative targets was composed of 6,755 nodes and 154,869 edges. (ii) The PPI network of CC-associated targets was made of 1,658 nodes and 26,392 edges. (iii) PPI network of HGWD against CC-associated targets was composed of 1,253 nodes and 22,705 edges. (iv) PPI network of key targets from iii. 311 nodes and 8,609 edges are shown. (v) PPI network of candidate core targets from iv. 107 nodes and 2,194 edges are shown. **(E)** The core targets were enriched in the representative biological processes (GO-BP) by using FUNRICH version 3 (*p* < 0.05) **(F)** The core targets were enriched in the representative signaling pathways (KEGG) by using DAVID v6.8 (*p* < 0.05).

According to our above results, HGWD could markedly suppress CC cell viability. From the perspective of TCM, most patients develop CC due to qi deficiency and blood stagnation, which is consistent with the main symptoms targeted by HGWD. Therefore, we further investigated the potential action mechanism by which HGWD treats CC. We downloaded and analysed three gene expression microarray datasets (GSE13471, GSE44076 and GSE33113) from human CC and the matching paracancerous tissues *via* the GEO database. We screened the datasets according to the conditions Fold change >2 and *p* < 0.01 and obtained 77 CC-related targets (Figures 4A–C and Supplementary Table S2). Then, we conducted PPI analyses of 243 probable drug targets of HGWD obtained previously using the BisoGenet plugin in Cytoscape and obtained 6,755 nodes and 154,869 edges. This plugin was used for PPI analysis of the mentioned 77 disease targets, resulting in 1,658 nodes and 26,392 edges. For precise prediction of potential core targets of HGWD in CC therapy, the CytoNCA plugin of Cytoscape was used to integrate the results of PPI analysis of disease and drug targets. After setting the thresholds (‘DC’>46, ‘DC’>78, ‘BC’>0.002, and ‘CC'’>0.47), we eventually excavated 107 potential core therapeutic targets (Figure 4D and Supplementary Table S3).

For prediction of biological processes and associated pathomechanisms related to the above core targets, FUNRICH v3 and DAVID v6.8 were used to conduct biological function (GO-Biological Process, GO-BP) and signaling pathway (KEGG) enrichment analysis, respectively. The obtained biological processes are closely related to “metabolism”, “signal transduction”, “apoptosis”, and “regulation of immune response” (Figure 4E). The enriched pathways were “pathways in cancer”, “viral carcinogenesis”, “PI3K-AKT signaling pathway”, and “MAPK signaling pathway” (Figure 4F). These findings imply that HGWD suppresses the viability of CC cells by impeding signaling pathways in cell proliferation, apoptosis, as well as metabolism.

### HGWD induces apoptosis and inhibits phosphorylation of the Akt/ERK signaling pathways in CC cells

**FIGURE 5:**
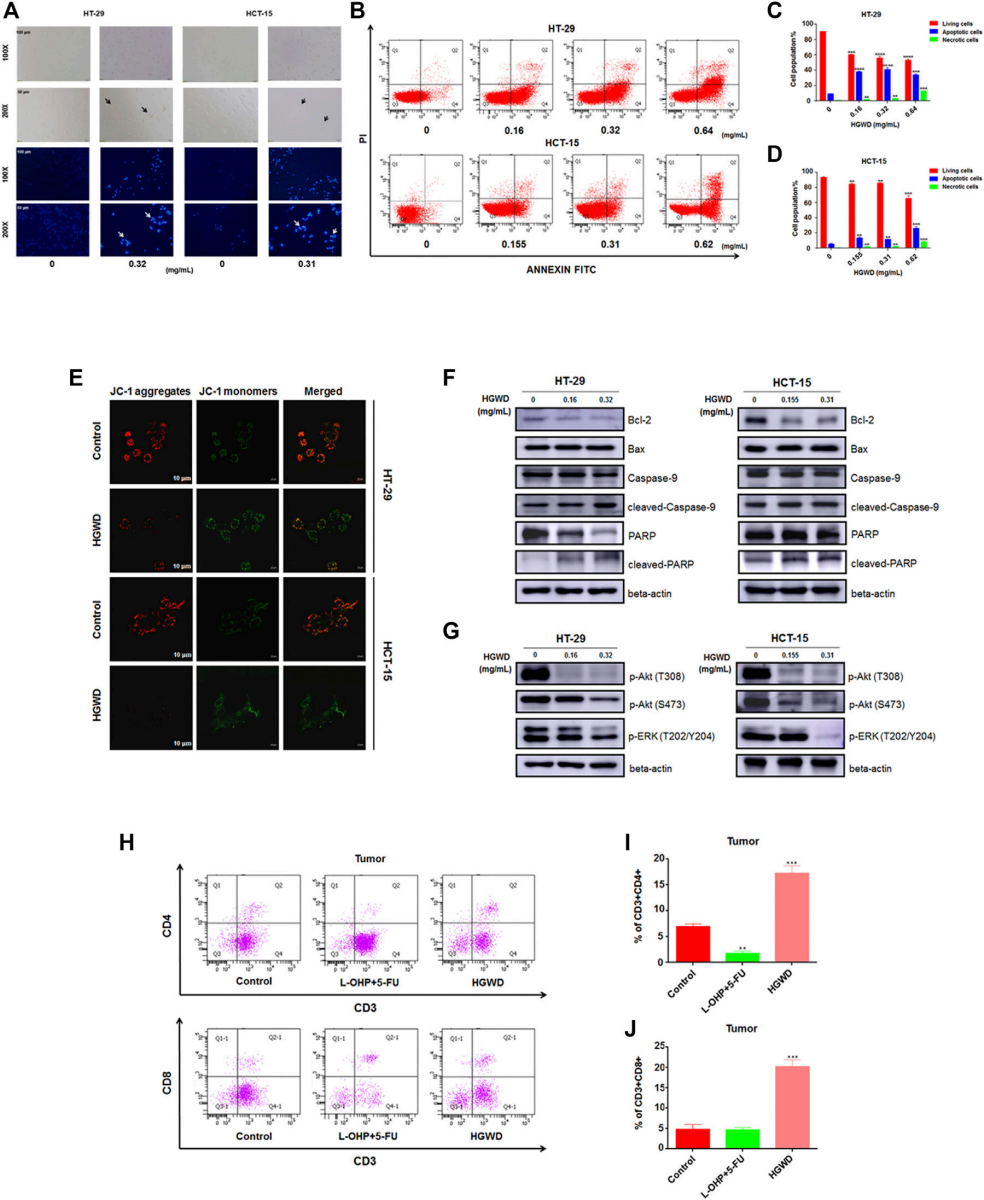
HGWD treatment enhanced apoptosis and down-regulated the levels of phosphorylation proteins in CC cell Akt and ERK signaling pathways. **(A)** CC cells morphologies were evaluated in white light and fluorescence field by inverted microscopy. **(B)** Induction of HT-29 as well as HCT-15 cell apoptosis after HGWD treatment for 24 h. Apoptotic processes were evaluated by flow cytometry. **(C,D)** Statistical assessments of the abundance of apoptotic cells in HT-29 and HCT-15 cells. **p < 0.01, ***p < 0.001 and ****p < 0.0001 **(E)** The mitochondrial membrane potential (MMP) decreased of HT-29 as well as HCT-15 cells 24 h after HGWD treatment **(F)** HT-29 as well as HCT-15 cells were treated with various HGWD doses for 24 h. After protein extraction, Bcl-2, pro-caspase-9, Bax, pro-PARP, cleaved-caspase-9, and cleaved-PARP levels were analyzed by WB, respectively. **(G)** HT-29 as well as HCT-15 cells were exposed to different HGWD doses for 24 h. After protein extractions, p-Akt (T308), p-ERK (T202/Y204) and p-Akt (S473) levels were respectively evaluated by WB **(H)** The abundance of the CD3^+^CD4^+^ and CD3^+^CD8^+^ T cells in xenografted CC tumors from different groups were detected by flow cytometry. **(I,J)** Statistical analyses of the abundance of the CD3^+^CD4^+^ and CD3^+^CD8^+^ T cells. **p < 0.01 and ***p < 0.001.

Then, we performed various functional assays to confirm the findings of the enrichment analyses. Relative to the control group, HGWD-treated cells displayed typical apoptotic characteristics such as cell shrinkage and wrinkling (Figure 5A). After Hoechst 33,342 staining showed that the nuclei of HGWD-treated cells had dense fragmented staining or dense staining (Figure 5A). Apoptotic cell population stained with Annexin V-FITC was markedly dose-dependently elevated after HGWD treatment (Figures 5B–D). In addition, JC-1 staining showed that MMP was significantly lower in cells treated with HGWD (Figure 5E). The WB results further demonstrated that HGWD dose-dependently promoted accumulation of the pro-apoptotic protein Bax, cleaved-Caspase-9 and cleaved-PARP and down-regulated expression of the preapoptotic protein Bcl-2 (Figure 5F and Supplementary Figure S1).

To evaluate the mechanisms through which HGWD suppresses CC cell growth, we assessed the key signaling pathways in cell proliferation as well as survival. Among these signaling pathways, with regards to KEGG enrichment results, MAPK and PI3K-AKT signaling pathways were selected. WB revealed that treatment with HGWD markedly inhibited the levels of phosphorylated key protein factors in these pathways, including p-ERK (T202/Y204) and p-Akt (T308 and S473) (Figure 5G and Supplementary Figure S2). These results indicated that HGWD-mediated induction of CC cells apoptosis and decreased of viability might be achieved through simultaneous inhibition of the phosphorylation of components of the Akt/ERK signaling pathways. In addition, we also examined tumor specimens preserved from previous *in vivo* experiments at the immunological level. Compared to positive drug and blank control groups, proportions of tumor-infiltrating CD3^+^CD4^+^ and CD3^+^CD8^+^ T cells were significantly increased after *in vivo* administration of HGWD for a period of time (Figures 5H–J). Intriguingly, the results indicate that HGWD exerted its therapeutic effect on CC by activating the animals’ immune systems, consistent with our biological process enrichment analysis.

### HGWD alters metabolic programming in CC-bearing mice

**FIGURE 6:**
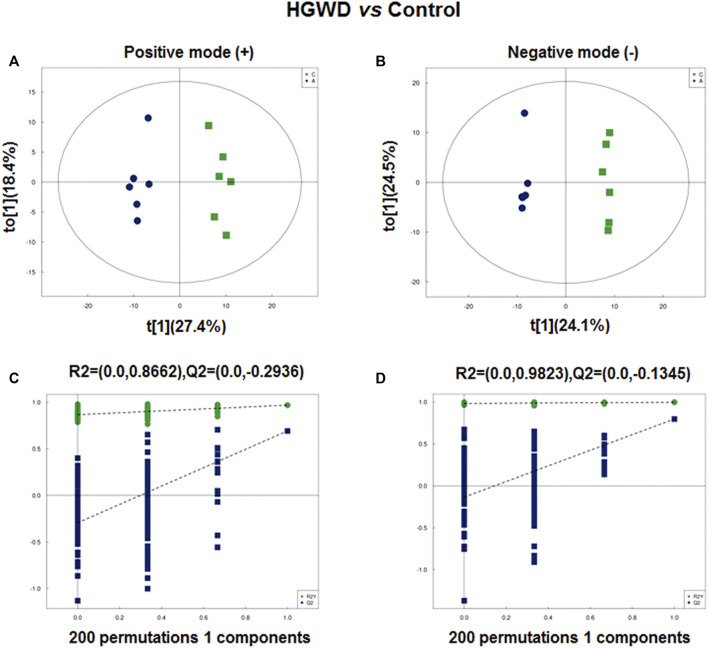
Plasma metabolomic analysis of xenografted CC tumors of C57BL/6 mice treated with HGWD. **(A,B)** Orthotopic partial least-squares discriminant analysis (OPLS-DA) score plots. A, positive ion; B, negative ion **(C,D)** Permutation tests for OPLS-DA score plots. C, positive ion; D, negative ion.

Since the enrichment results suggest that the potential function of HGWD in treatment of CC is closely related to metabolic processes, we performed *in vivo* non-targeted metabolomic analysis. We collected orbital blood from mice in the HGWD and the blank control group that were used in the previous *in vivo* pharmacodynamic experiments (six mice in each group). The orthogonal partial least squares discriminant analysis (OPLS-DA) model could distinguish the two groups of samples in both negative and positive ion modes (Figures 6A,B). To prevent the occurrence of overfitting during construction of the supervised model and to ensure the validity, we further verified the model using the permutation test. The results indicated that our model had no overfitting phenomenon (Figures 6C,D).

**FIGURE 7:**
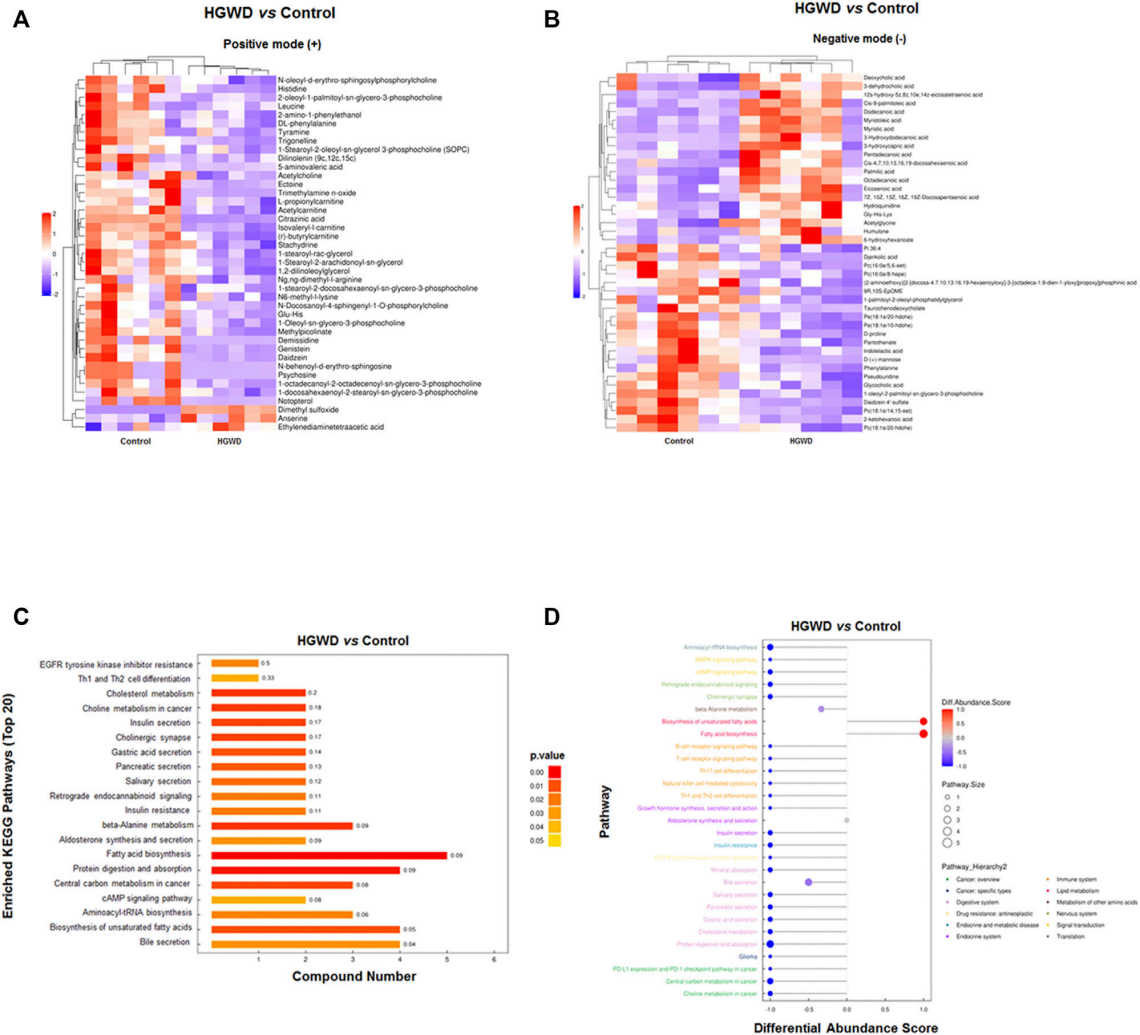
Altered plasma metabolites and related metabolic pathways in HGWD-treated xenografted CC tumors of C57BL/6 mice. **(A,B)** Heat plot of differentially expressed metabolites in HGWD-treated vs*.* control groups of mice. A, positive ion; B, negative ion. **(C)** The differentially expressed metabolites were enriched in the representative signaling pathways (KEGG). **(D)** Differentially expressed metabolites were also enriched in representative signaling pathways (KEGG, Pathway_Hierarchy two level).

After setting the multivariate and univariate statistical significance criteria (VIP>1 and *p*-value < 0.05), 42 and 41 differential metabolites were detected in the negative and positive ion modes (Tables 1, 2). Hierarchical clustering heatmaps of significant differential metabolites in the positive and negative ion modes are shown in Figures 7A,B, respectively. After administration of HGWD to tumor-bearing mice for a period of time, the significantly up-regulated metabolites identified in the positive ion mode mainly included dimethyl sulfoxide and anserine, while the significantly down-regulated metabolites mainly included psychosine and demissidine. Under the negative ion mode, the significantly up-regulated metabolites mainly included 3-hydroxydodecanoic acid and humulone, while the significantly down-regulated metabolites mainly included djenkolic acid and daidzein 4′-sulfate.

**TABLE 1:** Identified differentially expressed metabolites between HGWD-treated and control groups of mice (negative ion).

Metabolite	Rt (s)	m/z	VIP	Fold change (FC)	*p*-value
1-oleoyl-2-palmitoyl-sn-glycero-3-phosphocholine	138.47	794.57	2.27	0.72	0.0001
Pc(18:1e/14,15-eet)	38.92	868.60	1.75	0.52	0.0004
Daidzein 4′-sulfate	25.81	333.01	3.07	0.20	0.0009
Palmitic acid	50.47	255.23	12.34	1.43	0.0010
Myristic acid	51.10	227.20	5.54	2.93	0.0011
Dodecanoic acid	52.32	199.17	2.47	2.38	0.0022
7Z, 10Z, 13Z, 16Z, 19Z-Docosapentaenoic acid	47.68	329.25	2.44	2.06	0.0026
Octadecanoic acid	49.59	283.26	5.34	1.39	0.0035
Pc(16:0e/5,6-eet)	55.61	842.59	1.78	0.38	0.0036
6-hydroxyhexanoate	131.80	131.07	2.75	2.53	0.0043
Cis-9-palmitoleic acid	49.80	253.22	5.83	2.18	0.0045
Indolelactic acid	34.01	158.06	1.13	0.42	0.0057
Pe (18:1e/10-hdohe)	38.61	790.54	3.08	0.62	0.0062
Myristoleic acid	51.06	225.19	2.09	2.95	0.0062
Pantothenate	279.60	218.10	1.68	0.50	0.0068
2-ketohexanoic acid	37.02	129.06	1.47	0.56	0.0077
d-proline	315.24	114.06	1.23	0.56	0.0079
Phenylalanine	266.55	164.07	2.14	0.60	0.0082
(2-aminoethoxy)[2-[docosa-4.7.10.13.16.19-hexaenoyloxy]-3-[octadeca-1.9-dien-1-yloxy]propoxy]phosphinic acid	138.08	772.53	1.44	0.83	0.0082
Eicosenoic acid	47.82	309.28	2.14	2.00	0.0115
Pe (18:1e/20-hdohe)	140.86	790.54	3.95	0.74	0.0123
Pseudouridine	247.19	243.06	1.17	0.66	0.0149
D-(+)-mannose	117.83	179.03	1.63	0.25	0.0170
Pentadecanoic acid	50.51	241.22	1.04	1.52	0.0177
Gly-His-Lys	27.62	339.20	1.37	1.84	0.0178
Glycocholic acid	187.39	464.31	1.44	0.71	0.0189
Pc(16:0e/8-hepe)	140.44	840.58	2.36	0.75	0.0200
9R,10S-EpOME	57.03	295.23	1.95	0.58	0.0207
12s-hydroxy-5z,8z,10e,14z-eicosatetraenoic acid	54.39	319.23	2.85	3.23	0.0209
Taurochenodeoxycholate	162.68	498.29	3.38	0.30	0.0228
3-dehydrocholic acid	147.27	405.26	1.61	2.77	0.0251
3-Hydroxydodecanoic acid	92.45	215.16	1.23	3.70	0.0270
Cis-4,7,10,13,16,19-docosahexaenoic acid	48.82	327.23	5.40	1.83	0.0302
Djenkolic acid	25.26	253.05	2.59	0.18	0.0320
Acetylglycine	430.00	233.08	3.16	1.38	0.0345
Pi 36:4	196.77	857.52	7.16	0.58	0.0375
Pc(18:1e/20-hdohe)	137.33	892.60	1.43	0.79	0.0402
Deoxycholic acid	160.21	391.28	1.50	1.78	0.0411
Humulone	178.01	361.20	1.32	3.50	0.0420
Hydroquinidine	28.01	325.18	1.16	1.72	0.0433
1-palmitoyl-2-oleoyl-phosphatidylglycerol	38.26	747.52	1.68	0.78	0.0449
3-hydroxycapric acid	106.62	187.13	1.15	1.83	0.0471

**TABLE 2:** Identified differentially expressed metabolites between HGWD-treated and control groups of mice (positive ion).

Metabolite	Rt (s)	m/z	VIP	Fold change (FC)	*p*-value
Citrazinic acid	64.77	156.04	2.10	0.03	<0.0001
Dimethyl sulfoxide	59.53	79.02	31.12	475.49	<0.0001
Isovaleryl-l-carnitine	240.81	246.17	1.79	0.42	<0.0001
Acetylcarnitine	307.37	204.12	6.35	0.59	0.0001
(r)-butyrylcarnitine	259.09	232.15	4.28	0.53	0.0002
Genistein	25.59	271.06	1.09	0.23	0.0009
1-stearoyl-rac-glycerol	197.00	341.30	1.14	0.57	0.0011
Trigonelline	295.58	138.05	4.26	0.37	0.0011
Daidzein	26.40	255.06	2.47	0.15	0.0013
Trimethylamine n-oxide	332.97	76.08	1.55	0.08	0.0013
Methylpicolinate	222.65	138.05	1.14	0.32	0.0019
1-Stearoyl-2-arachidonoyl-sn-glycerol	196.37	627.53	7.92	0.44	0.0026
1-octadecanoyl-2-octadecenoyl-sn-glycero-3-phosphocholine	139.16	810.60	9.24	0.71	0.0027
1-Oleoyl-sn-glycero-3-phosphocholine	189.35	544.34	10.89	0.67	0.0055
Histidine	523.50	156.08	2.19	0.42	0.0076
Ectoine	339.01	143.08	1.40	0.17	0.0082
N-behenoyl-d-erythro-sphingosine	79.55	622.61	1.69	0.01	0.0099
Psychosine	86.30	444.33	1.69	0.01	0.0103
Demissidine	58.25	400.38	1.54	0.01	0.0105
1,2-dilinoleoylglycerol	197.66	599.50	3.32	0.57	0.0108
N-oleoyl-d-erythro-sphingosylphosphorylcholine	177.44	729.59	1.07	0.83	0.0126
2-oleoyl-1-palmitoyl-sn-glycero-3-phosphocholine	141.06	782.57	6.62	0.82	0.0130
Notopterol	53.38	187.12	1.43	0.05	0.0131
Anserine	418.26	241.13	1.04	1.76	0.0133
Glu-His	26.34	285.07	1.14	0.29	0.0158
Dilinolenin (9c,12c,15c)	31.19	613.48	1.03	0.41	0.0199
L-propionylcarnitine	282.57	218.14	1.81	0.65	0.0206
1-stearoyl-2-docosahexaenoyl-sn-glycero-3-phosphocholine	72.72	834.59	1.09	0.70	0.0208
Tyramine	297.62	120.08	1.71	0.44	0.0234
Acetylcholine	379.58	146.12	1.19	0.69	0.0256
2-amino-1-phenylethanol	266.55	120.08	2.00	0.58	0.0270
Stachydrine	276.09	144.10	2.02	0.63	0.0272
N6-methyl-l-lysine	534.52	161.13	1.62	0.44	0.0305
N-Docosanoyl-4-sphingenyl-1-O-phosphorylcholine	174.24	809.65	1.46	0.77	0.0351
1-docosahexaenoyl-2-stearoyl-sn-glycero-3-phosphocholine	137.92	834.60	4.48	0.82	0.0407
Ethylenediaminetetraacetic acid	449.72	293.10	35.96	1.40	0.0418
1-Stearoyl-2-oleoyl-sn-glycerol 3-phosphocholine (SOPC)	137.92	832.58	2.58	0.83	0.0434
DL-phenylalanine	266.53	166.09	1.18	0.64	0.0442
Ng,ng-dimethyl-l-arginine	499.93	203.15	1.19	0.48	0.0447
5-aminovaleric acid	341.11	118.09	2.37	0.20	0.0463
Leucine	274.86	132.10	1.39	0.46	0.0493

Subsequently, we conducted the KEGG enrichment analysis of significant differential metabolites obtained in the previous step. The results showed that these metabolites are closely related to “Protein digestion and absorption”, “Cholesterol metabolism”, “beta-alanine metabolism”, “Fatty acid biosynthesis”, and “Choline metabolism in cancer” (Figure 7C). Analysis of the overall changes in KEGG metabolic pathways revealed that the above significant differential metabolites were mainly closely related to “cancer”, “digestive system”, “metabolism of other amino acids”, “immune system”, “lipid metabolism”, and “signal transduction” (such as MAPK signaling pathway) (Figure 7D).

### CYP2E1 is a potential key target of HGWD in CC treatment

**FIGURE 8:**
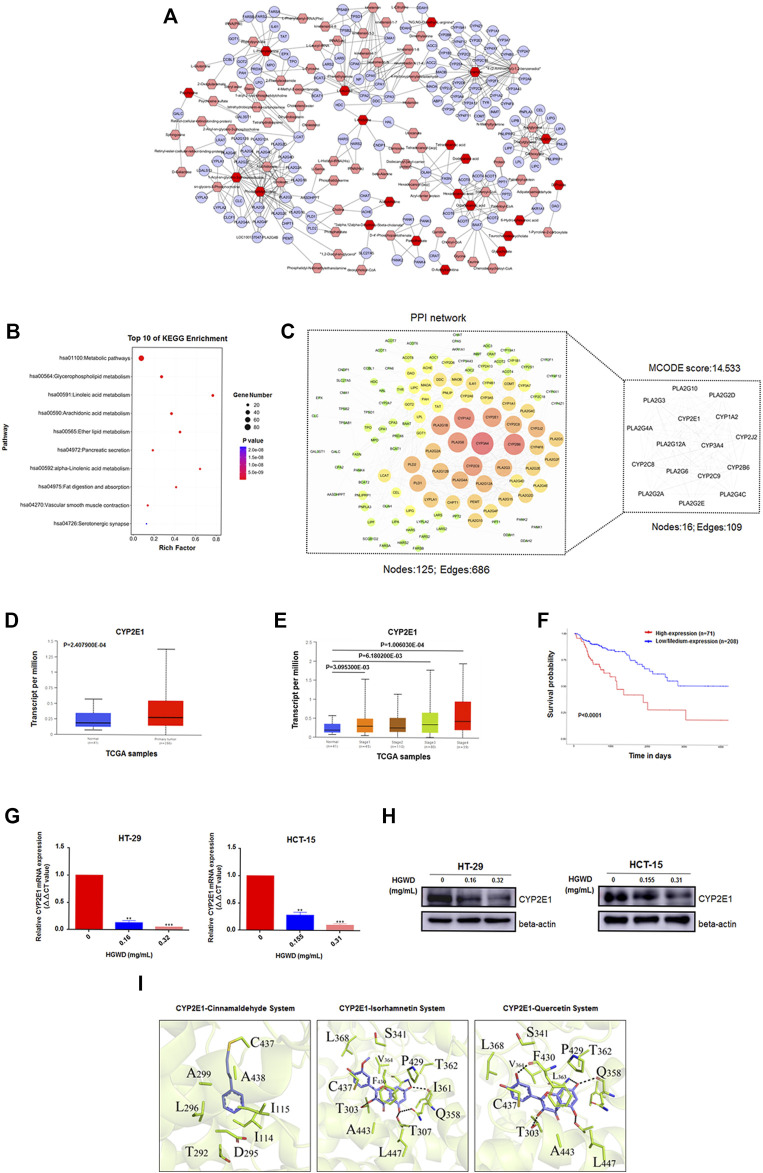
CYP2E1 is a candidate key target of HGWD in the treatment of CC **(A)** The network of potential biomarkers of HGWD for the anti-CC effect. It was constructed using MetScape, and the nodes represented related metabolites and targets regulated by significantly metabolites, and edges represented biochemical reactions. **(B)** The above targets regulated by metabolites were enriched in representative signaling pathways (KEGG) using DAVID v6.8 (*p* < 0.05). **(C)** The PPI network of above targets regulated by metabolites was established, and screened the candidate key targets by MCODE. **(D–F)** CYP2E1 was a potential key target in CC by UALCAN online database screening. **(G,H)** The expression levels of CYP2E1 were verified by qRT-PCR and WB in HGWD-treated and control CC cells, respectively. **p < 0.01 and ***p < 0.001 **(I)** Results of molecular docking studies of three ingredients (cinnamaldehyde, isorhamnetin and quercetin) in the active sites of CYP2E1.

To further understand the relationship among the 83 significant differential metabolites, we first mapped the KEGG IDs of the differential metabolites using MetaboAnalyst software and obtained a total of 21 known IDs corresponding to the differential metabolites. We then imported the ID information obtained in the previous step into the system using MetScape software and thus constructed and visualized a systematic network of differential metabolites and regulatory targets. Eventually, a total of 134 potential regulatory targets were obtained (Figure 8A). Next, the KEGG enrichment results indicated that these targets are closely related to “metabolic pathways”, “linoleic acid metabolism” and “glycerophospholipid metabolism” (Figure 8B). We also performed PPI assessments of the 134 targets *via* the STRING database (https://cn.string-db.org/) and visualized the network using Cytoscape software. Subsequently, we mined and analysed the core subnetworks of the above PPI network using the MCODE plugin in Cytoscape software; this eventually resulted in the acquisition of the first-ranked core subnetwork (MCODE score: 14.533) containing 16 potential core targets (Figure 8C). We then further screened CYP2E1 using the TCGA module of the public online database UALCAN. CYP2E1 expression was markedly high in CC tissues, relative to paracancerous tissues. Moreover, CYP2E1 levels were higher in CC patients with terminal pathological stage (Normal vs. Stage 4, *p* = 1.006030E-04). Meanwhile, CC patients with elevated CYP2E1 levels exhibited markedly poor overall survival (OS) and prognosis than low levels (Figures 8D–F). Furthermore, HGWD reduced the mRNA and protein expressions of intracellular CYP2E1, suggesting that CYP2E1 is a potential key target of HGWD in CC therapy (Figures 8G,H and Supplementary Figure S3).

Since the herb HQ and GZ in HGWD generally plays an important role in delivery of its pharmacological effect, we next set out to identify the key in-blood ingredient in HQ and GZ of HGWD using the UPLC-QTOF-MS method. Nine key ingredients from the mouse plasma samples were identified, including quercetin (t_R_: 6.93 min), isorhamnetin (t_R_: 7.33 min), formononetin (t_R_: 8.83 min), kaempferol (t_R_: 7.92 min), astragaloside III (t_R_: 8.36 min), astragaloside IV (t_R_: 9.10 min), ent-epicatechin (t_R_: 1.08 min), taxifolin (t_R_: 6.55 min) and cinnamaldehyde (t_R_: 7.89 min), which partially validated the key ingredients previously selected based on the ADME-related characteristics and literature searching (Table 3). Next, the docking efficiency between the above nine key in-blood ingredients and the key target CYP2E1 were predicted, among which the binding affinities of cinnamaldehyde, isorhamnetin and quercetin with CYP2E1 (energy: −9.61, −8.86 and −8.81 kcal/mol, respectively) were greater than those between the other ingredients and target (Figure 8I and Table 4). However, further experimental verification is warranted.

**TABLE 3:** Identification of in-blood ingredient in HGWD by UPLC-QTOF-MS data.

No.	t_R_ (min)	Molecular formula	Selected ion	Theoretical	Experimental	MS/MS fragmentions	Compounds
1	6.93	C_15_H_10_O_7_	[M-H]^-^	302.23	301.03	273.00	Quercetin
2	7.33	C_16_H_12_O_7_	[M-H]^-^	316.26	315.05	151.33, 125.08	Isorhamnetin
3	8.83	C_16_H_12_O_4_	[M-H]^-^	268.26	267.07	251.90, 223.00	Formononetin
4	7.92	C_15_H_10_O_6_	[M-H]^-^	286.24	285.04	195.60, 186.53	Kaempferol
5	8.36	C_41_H_68_O_14_	[M-H]^-^	785.00	783.45	783.45, 223.06	Astragaloside III
6	9.10	C_41_H_68_O_14_	[M-H]^+^	785.00	785.47	473.50	Astragaloside IV
7	1.08	C_15_H_14_O_6_	[M-H]^-^	290.27	289.07	139.04, 123.04	Ent-Epicatechin
8	6.55	C_15_H_12_O_7_	[M-H]^-^	304.25	303.05	192.91, 134.74	Taxifolin
9	7.89	C_9_H_8_O	[M-H]^+^	132.16	133.06	115.05, 105.07	Cinnamaldehyde

**TABLE 4:** Results of molecular docking studies of nine ingredients in the active sites of CYP2E1 performed using Autodock 4.2.

Target	Ingredients	Binding energy (kcal/mol)	Target	Ingredients	Binding energy (kcal/mol)
CYP2E1(3E4E)	Quercetin	−8.81	CYP2E1(3E4E)	Astragaloside IV	−8.30
	Isorhamnetin	−8.86		Ent-Epicatechin	−7.85
	Formononetin	−7.91		Taxifolin	−8.54
	Kaempferol	−8.51		Cinnamaldehyde	−9.61
	Astragaloside III	−8.33			

## Discussion

In 2007, Hopkins proposed the concept of network pharmacology for the first time and predicted that it would become “the next paradigm in drug discovery” (Hopkins, 2007). In the same year, Li proposed the construction of a research framework of TCM prescriptions based on biological networks (Li, 2007). Indeed, in recent years, network pharmacology has provided new methods for studies in the field of TCM, especially in new TCM development and drug repositioning (Chong and Sullivan, 2007). At present, we have adopted this emerging technology to carry out a series of important works in the TCM repositioning (Pan et al., 2019; Pan et al., 2020; Pan et al., 2021). In this study, we found for the first time that HGWD has a potentially therapeutic effect on CC. This finding reflects the potential of HGWD for new clinical use. In the TCM theory, CC patients is often qi deficiency in spleen and stomach. Qi deficiency leads to blood stagnation, while blood stasis blockage and accumulation of dampness toxin predispose to tumor formation. HGWD has the effect of enhancing qi and blood, warming meridians and dredging collaterals, which is consistent with the main syndrome treated by HGWD. Therefore, HGWD can be used to treat CC patients with qi deficiency and blood stagnation.

Our *in vivo* assay showed that orally administered HGWD markedly suppressed the proliferation and viability of CC cells in tumor-bearing C57BL/6 mice. In addition, HGWD exerts its therapeutic effect on CC by regulating the animals’ immune systems. Meanwhile, treatment of CC with HGWD could affect several biological functions and signaling pathways, mainly including regulation of metabolic reprogramming, apoptosis, PI3K/AKT and MAPK/ERK signaling pathways. Some ingredients of five CHMs in HGWD have potential anti-tumor effects. For instance, astragaloside IV, the main component of Huangqi, inhibits invasion as well as metastasis in SiHa cervical cancer cells through TGF-β1-related MAPK as well as PI3K signaling pathways (Zhang et al., 2019). Meanwhile, Astragaloside IV could inhibit cell proliferation of CC cell lines (SW620 and HCT116) through down-regulation of B7-H3 (Wang et al., 2018), and it could also exert anti-CC effect by re-educating tumor-associated macrophage (Liu et al., 2020). Cinnamic acid, a key component of Guizhi, induces apoptosis and reduces melanoma cell proliferation, while derivatives of cinnamic acid induce apoptosis of CC and cervical cancer cells (Niero and Machado-Santelli, 2013; Anantharaju et al., 2017). Cinnamaldehyde, another important ingredient of Guizhi, could affect the biological behavior of human CC cells (SW480, HCT116 and LoVo), and induced apoptosis *via* inhibition of the PI3K/Akt signaling pathway (Li et al., 2016). In addition, it has been reported that paeoniflorin, an active ingredient in Shaoyao, suppresses the proliferations of pancreatic cancer cells and endometrial carcinoma cells by dysregulating the MAPK/ERK signaling pathway (Yang et al., 2016; Zhang et al., 2017). Intriguingly, in this study, although we did not examine the effects regarding the role of HGWD on the inhibition of metastasis in CC, some components of five CHMs in HGWD have potential anti-metastasis effects. For instance, quercetin, the key component of Huangqi, could inhibit metastasis of cancer cells by blocking Akt/mTOR/c-Myc signaling pathway to suppress RPS19-activated EMT signaling (Chen et al., 2018b). Meanwhile, cinnamaldehyde could also decrease the metastasis of osteosarcoma by down-regulating the FAK signaling pathway (Chu et al., 2022).

The most exciting new finding of this study is that the metabolic level changed significantly in tumor-bearing C57BL/6 mice after treatment with HGWD for a period of time. At present, an increasing amount of evidence shows that cancer is a special metabolism-associated disease (Phan et al., 2014; Wang et al., 2020b; Qian et al., 2021). *In vivo* metabolic reprogramming is one of the important markers of tumorigenesis. Cancer cells obtain growth advantages or escape various forms of cell death, such as apoptosis, through abnormal metabolic regulation (Hanahan and Weinberg, 2011). Our results showed that HGWD mediated marked changes in abundance of many metabolites in CC-bearing mice, indicating that HGWD could target metabolic processes. Furthermore, most of the significantly metabolites in HGWD group were closely associated with lipid and cholesterol metabolism. Recent relevant studies indicate that fatty acid biosynthesis pathway, as a vital process in lipid metabolism, is crucial to the survival of tumor cells, therefore, limiting the fatty acid biosynthesis pathway may provide a strategy for cancer treatment (Currie et al., 2013). Meanwhile, cholesterol metabolism produces essential membrane components and metabolites with multiple biological functions. In the tumor microenvironment, internal and external factors that are relevant to cellular activity may reprogram cholesterol metabolism, thereby promoting tumor development and progression. However, relevant clinical studies also show that controlling cholesterol metabolism can inhibit tumorigenesis (Huang et al., 2020).

For network pharmacology studies, especially on Chinese herbal decoction, the serum metabolomics-based network pharmacology strategy can integrate more comprehensive targets for prediction of therapeutical targets as well as their interactions. Therefore, we further constructed a network of anti-CC metabolites–regulatory targets using MetScape based on the significant differential metabolites regulated by HGWD *in vivo.* We found that CYP2E1 is a potential key target in CC treatment. The cytochrome P450 family (CYPs) is a superfamily of proteins that contain haeme as a cofactor. CYPs have a vital role in metabolism of various exogenous substances, and it also closely relate to chemical carcinogenesis through their ability to activate or inactivate carcinogens, thus affecting the tumorigenesis (Ortega-Ugalde et al., 2019; Qu et al., 2019). CYP2E1 is the main P450 enzyme involved in ethanol metabolism, and its expression is also closely contacted with tumor diseases. Overexpressed CYP2E1 was shown to enhance the proliferation as well as invasion of MGC-803 gastric cancer cells and inhibits their apoptosis while up-regulating the levels of intracellular p-Akt, p-mTOR and p-P70S6K (Wang et al., 2020c). Another study showed that the RsaI/PstI and 96-bp insertion polymorphisms of CYP2E1 may be closely related to the risk of developing CC (Jiang et al., 2013). Furthermore, CYP2E1 is also closely related to the tumor immune microenvironment because its expression correlates strongly with the number of infiltrating monocytes and regulatory T cells (Treg) (Ye et al., 2021). According to a study that demonstrated a negative correlation between CYP2E1 and immune checkpoints, changes in the expression level of CYP2E1 in glioma were related to the immunosuppressive characteristics of the tumor microenvironment (Wang et al., 2019). As a target with lipid metabolism process, CYP2E1 expression is also closely related to the immune microenvironment. Thus, the application of CYP2E1 as a potential therapeutic target has shown a good prospect for future treatment.

Admittedly, our study has some limitations and needs further improvement. First, the role and mechanism of CYP2E1 in colon cancer were not further explored in this study, for example, cell functional assays need to be performed to investigate the effects of CYP2E1 on the proliferation, migration, and invasion of CC cells. Meanwhile, the active compounds and mechanisms of action of HGWD on CYP2E1 warrant further investigation. In addition, although the predicted binding affinity of cinnamaldehyde, isorhamnetin and quercetin to CYP2E1 (-9.61, -8.86 and -8.81 kcal/mol, respectively) are good, experimental validation such as SPR and ITC assays should be further conducted.

## Conclusion

Our study show that HGWD regulates major biological functions as well as processes in CC cells survival, including induction of apoptosis and participation in immune regulation. The regulation of these by HGWD may be due to simultaneous suppression of various molecular signaling pathways, such as Akt and ERK pathways. In addition, metabolomic analysis showed that HGWD plays an important anti-CC role by regulating important metabolic pathways that are closely associated with tumorigenesis, such as lipid metabolism and cholesterol metabolism, and CYP2E1 is a potential therapeutic target of HGWD in CC treatment. In short, our study used a comprehensive strategy that combined serum metabolomics with TCM network pharmacology, thereby providing a scientific basis for elucidation of the anti-CC mechanism of HGWD.

## Data Availability

The original contributions presented in the study are included in the article/Supplementary Material, further inquiries can be directed to the corresponding authors.

## Supplementary material

Supplementary Figure S1 The original western blot images for Bcl-2, Bax, pro-caspase-9, cleaved-caspase-9, pro-PARP, cleaved-PARP and beta-actin.

Supplementary Figure S2 The original western blot images for p-Akt (T308), p-Akt (S473), p-ERK (T202/Y204) and beta-actin.

Supplementary Figure S3 The original western blot images for CYP2E1 and beta-actin.

## Abbreviations

HGWD, Huangqi Guizhi Wuwu Decoction; CC, Colon cancer; TCM, Traditional Chinese medicine; CHM, Chinese herb medicine; PPI, Protein-protein interaction; DC, Degree centrality; BC, Betweenness centrality; CC', Closeness centrality; FBS, Foetal bovine serum; L-OHP, oxaliplatin; 5-FU, 5-fluorouracil; OPLS-DA, Orthogonal partial least squares discriminant analysis; CYP2E1, Cytochrome P450 family 2 subfamily E member 1; VIP, Variable importance in the projection.

## References

[B1] Anantharaju P. Reddy D. Padukudru M. Kumari-Chitturi C. Vimalambike M. Madhunapantula S. (2017). Induction of colon and cervical cancer cell death by cinnamic acid derivatives is mediated through the inhibition of Histone Deacetylases (HDAC). PLoS One 12, e0186208. 10.1371/journal.pone.0186208 29190639 PMC5708809

[B2] Brody H. (2015). Beauty. Nature 521, S1. 10.1038/526S1a 26444366

[B3] Center M. Jemal A. Smith R. Ward E. (2009). Worldwide variations in colorectal cancer. Ca. Cancer J. Clin. 59, 366–378. 10.3322/caac.20038 19897840

[B4] Chen K. Hsu W. Ho J. Lin C. Chu C. Kandaswami C. (2018a). Flavonoids luteolin and quercetin inhibit RPS19 and contributes to metastasis of cancer cells through c-Myc reduction. J. Food Drug Anal. 26, 1180–1191. 10.1016/j.jfda.2018.01.012 29976410 PMC9303038

[B5] Chong C. Sullivan D. (2007). New uses for old drugs. Nature 448, 645–646. 10.1038/448645a 17687303

[B6] Chu S. Hsieh Y. Hsu L. Lin C. Lai Y. Chen P. (2022). Cinnamaldehyde decreases the invasion and u-PA expression of osteosarcoma by down-regulating the FAK signalling pathway. Food Funct. 13, 6574–6582. 10.1039/d2fo00634k 35678522

[B7] Chen C. Liao H. H. Chiang J. H. Wu M. Y. Chen B. C. Chang C. (2018b). Complementary Chinese herbal medicine therapy improves survival of patients with pancreatic cancer in taiwan: A nationwide population-based cohort study. Integr. Cancer Ther. 17, 411–422. 10.1177/1534735417722224 28774207 PMC6041895

[B8] Currie E. Schulze A. Zechner R. Walther T. Farese R. (2013). Cellular fatty acid metabolism and cancer. Cell Metab. 18, 153–161. 10.1016/j.cmet.2013.05.017 23791484 PMC3742569

[B9] Edwards B. Ward E. Kohler B. Eheman C. Zauber A. Anderson R. (2010). Annual report to the nation on the status of cancer, 1975-2006, featuring colorectal cancer trends and impact of interventions (risk factors, screening, and treatment) to reduce future rates. Cancer 116, 544–573. 10.1002/cncr.24760 19998273 PMC3619726

[B10] Guijas C. Montenegro-Burke J. Warth B. Spilker M. Siuzdak G. (2007). Metabolomics activity screening for identifying metabolites that modulate phenotype. Nat. Biotechnol. 36, 316–320. 10.1038/nbt.4101 PMC5937131 29621222

[B11] Hopkins A. (2007). Network pharmacology. Nat. Biotechnol. 25, 1110–1111. 10.1038/nbt1007-1110 17921993

[B12] Hanahan D. Weinberg R. (2011). Hallmarks of cancer: The next generation. Cell 144, 646–674. 10.1016/j.cell.2011.02.013 21376230

[B13] Hopkins A. (2008). Network pharmacology: The next paradigm in drug discovery. Nat. Chem. Biol. 4, 682–690. 10.1038/nchembio.118 18936753

[B14] Huang B. Song B. Xu C. (2020). Cholesterol metabolism in cancer: Mechanisms and therapeutic opportunities. Nat. Metab. 2, 132–141. 10.1038/s42255-020-0174-0 32694690

[B15] Jiang O. Zhou R. Wu D. Liu Y. Wu W. Cheng N. (2013). CYP2E1 polymorphisms and colorectal cancer risk: A HuGE systematic review and meta-analysis. Tumour Biol. 34, 1215–1224. 10.1007/s13277-013-0664-8 23355335

[B16] Li J. Teng Y. Liu S. Wang Z. Chen Y. Zhang Y. (2016). Cinnamaldehyde affects the biological behavior of human colorectal cancer cells and induces apoptosis via inhibition of the PI3K/Akt signaling pathway. Oncol. Rep. 35, 1501–1510. 10.3892/or.2015.4493 26677144

[B17] Li S. (2007). [Framework and practice of network-based studies for Chinese herbal formula]. J. Chin. Integr. Med. 5, 489–493. 10.3736/jcim20070501 17854545

[B18] Liang L. Wei X. Feng M. Zhu L. Yu J. Yang G. (2020). Huangqi Guizhi Wuwu decoction for treating cervical radiculopathy: A systematic review and meta-analysis of randomized controlled trials. Medicine 99, e19137. 10.1097/MD.0000000000019137 32049834 PMC7035008

[B19] Liu F. Ran F. He H. Chen L. (2020). Astragaloside IV exerts anti-tumor effect on murine colorectal cancer by re-educating tumor-associated macrophage. Arch. Immunol. Ther. Exp. 68, 33. 10.1007/s00005-020-00598-y 33095374

[B20] Liu W. Shi L. Wan Q. Wu Y. Huang D. Ou J. (2021). Huangqi Guizhi Wuwu Decoction attenuates Podocyte cytoskeletal protein damage in IgA nephropathy rats by regulating AT1R/Nephrin/c-Abl pathway. Biomed. Pharmacother. 142, 111907. 10.1016/j.biopha.2021.111907 34339916

[B21] Niero E. Machado-Santelli G. (2013). Cinnamic acid induces apoptotic cell death and cytoskeleton disruption in human melanoma cells. J. Exp. Clin. Cancer Res. 32, 31. 10.1186/1756-9966-32-31 23701745 PMC3667113

[B22] Ortega-Ugalde S. Boot M. Commandeur J. Jennings P. Bitter W. Vos J. (2019). Function, essentiality, and expression of cytochrome P450 enzymes and their cognate redox partners in *Mycobacterium tuberculosis*: Are they drug targets? Appl. Microbiol. Biotechnol. 103, 3597–3614. 10.1007/s00253-019-09697-z 30810776 PMC6469627

[B23] Pan B. Fang S. Zhang J. Pan Y. Liu H. Wang Y. (2020). Chinese herbal compounds against SARS-CoV-2: Puerarin and quercetin impair the binding of viral S-protein to ACE2 receptor. Comput. Struct. Biotechnol. J. 18, 3518–3527. 10.1016/j.csbj.2020.11.010 33200026 PMC7657012

[B24] Pan B. Shi X. Ding T. Liu L. (2019). Unraveling the action mechanism of *polygonum cuspidatum* by a network pharmacology approach. Am. J. Transl. Res. 11, 6790–6811. 31814888 PMC6895524

[B25] Pan B. Wang Y. Wu C. Jia J. Huang C. Fang S. (2021). A mechanism of action study on Danggui Sini decoction to discover its therapeutic effect on gastric cancer. Front. Pharmacol. 11, 592903. 10.3389/fphar.2020.592903 33505310 PMC7830678

[B26] Pan B. Zang J. He J. Wang Z. Liu L. (2018). Add-on therapy with Chinese herb medicine bo-Er-ning capsule (BENC) improves outcomes of gastric cancer patients: A randomized clinical trial followed with bioinformatics-assisted mechanism study. Am. J. Cancer Res. 8, 1090–1105. 30034946 PMC6048393

[B27] Petrelli F. Labianca R. Zaniboni A. Lonardi S. Galli F. Rulli E. (2020). Assessment of duration and effects of 3 vs 6 Months of adjuvant chemotherapy in high-risk stage II colorectal cancer: A subgroup Analysis of the tosca randomized clinical trial. JAMA Oncol. 6, 547–551. 10.1001/jamaoncol.2019.6486 32053133 PMC7042800

[B28] Phan L. Yeung S. Lee M. (2014). Cancer metabolic reprogramming: Importance, main features, and potentials for precise targeted anti-cancer therapies. Cancer Biol. Med. 11, 1–19. 10.7497/j.issn.2095-3941.2014.01.001 24738035 PMC3969803

[B29] Qian F. Zhang M. Yin L. Lei Q. (2021). Cancer metabolism and dietary interventions. Cancer Biol. Med. 19, 163–174. 10.20892/j.issn.2095-3941.2021.0461 PMC8832959 34931768

[B30] Qu R. Li X. Quan X. Xia L. Fang X. Li H. (2019). Polymorphism in CYP24A1 is associated with lung cancer risk: A case-control study in Chinese female nonsmokers. DNA Cell Biol. 38, 243–249. 10.1089/dna.2018.4510 30724597

[B31] Ru J. Li P. Wang J. Zhou W. Li B. Huang C. (2014). Tcmsp: A database of systems pharmacology for drug discovery from herbal medicines. J. Cheminform. 6, 13. 10.1186/1758-2946-6-13 24735618 PMC4001360

[B32] Sanoff H. Sargent D. Campbell M. Morton R. Fuchs C. Ramanathan R. (2008). Five-year data and prognostic factor analysis of oxaliplatin and irinotecan combinations for advanced colorectal cancer: N9741. J. Clin. Oncol. 26, 5721–5727. 10.1200/JCO.2008.17.7147 19001325 PMC2645101

[B33] Shannon P. Markiel A. Ozier O. Baliga N. Wang J. Ramage D. (2003). Cytoscape: A software environment for integrated models of biomolecular interaction networks. Genome Res. 13, 2498–2504. 10.1101/gr.1239303 14597658 PMC403769

[B34] Tang J. Liu B. Ma K. (2008). Traditional Chinese medicine. Lancet 372, 1938–1940. 10.1016/S0140-6736(08)61354-9 18930523

[B35] Tariq K. Ghias K. (2016). Colorectal cancer carcinogenesis: A review of mechanisms. Cancer Biol. Med. 13, 120–135. 10.28092/j.issn.2095-3941.2015.0103 27144067 PMC4850121

[B36] Wang L. Fan Y. Xin P. Zhao Y. Deng H. Jia B. (2020). The efficacy and safety of Huangqi Guizhi Wuwu decoction for rheumatoid arthritis: A protocol for systematic review and meta-analysis. Medicine 99, e22011. 10.1097/MD.0000000000022011 32899051 PMC7478548

[B37] Wang R. Chen X. Zhang W. Jiang F. Liu M. Shen X. (2020). CYP2E1 changes the biological function of gastric cancer cells via the PI3K/Akt/mTOR signaling pathway. Mol. Med. Rep. 21, 842–850. 10.3892/mmr.2019.10890 31974627 PMC6947835

[B38] Wang S. Mou J. Cui L. Wang X. Zhang Z. (2018). Astragaloside IV inhibits cell proliferation of colorectal cancer cell lines through down-regulation of B7-H3. Biomed. Pharmacother. 102, 1037–1044. 10.1016/j.biopha.2018.03.127 29710520

[B39] Wang X. Guo G. Guan H. Yu Y. Lu J. Yu J. (2019). Challenges and potential of PD-1/PD-L1 checkpoint blockade immunotherapy for glioblastoma. J. Exp. Clin. Cancer Res. 38, 87. 10.1186/s13046-019-1085-3 30777100 PMC6380009

[B40] Wang Z. Jiang Q. Dong C. (2020). Metabolic reprogramming in triple-negative breast cancer. Cancer Biol. Med. 17, 44–59. 10.20892/j.issn.2095-3941.2019.0210 32296576 PMC7142847

[B41] Wei X. Chen X. Shu P. Jiang Z. Wu X. Zou X. (2020). Study on efficacy and safety of Huangqi Guizhi Wuwu decoction treatment for oxaliplatin induced peripheral neurotoxicity: A protocol for a randomized, controlled, double-blind, multicenter trial. Medicine 99, e19923. 10.1097/MD.0000000000019923 32481364 PMC12245263

[B42] Yang N. Cui H. Han F. Zhang L. Huang T. Zhou Y. (2016). Paeoniflorin inhibits human pancreatic cancer cell apoptosis via suppression of MMP-9 and ERK signaling. Oncol. Lett. 12, 1471–1476. 10.3892/ol.2016.4761 27446455 PMC4950818

[B43] Ye L. Xu Y. Wang L. Zhang C. Hu P. Tong S. (2021). Downregulation of CYP2E1 is associated with poor prognosis and tumor progression of gliomas. Cancer Med. 10, 8100–8113. 10.1002/cam4.4320 34612013 PMC8607268

[B44] Zhang J. Wang F. Wang H. Wang Y. Wu Y. Xu H. (2017). Paeoniflorin inhibits proliferation of endometrial cancer cells via activating MAPK and NF-κB signaling pathways. Exp. Ther. Med. 14, 5445–5451. 10.3892/etm.2017.5250 29285074 PMC5740769

[B45] Zhang L. Zhou J. Qin X. Huang H. Nie C. (2019). Astragaloside IV inhibits the invasion and metastasis of SiHa cervical cancer cells via the TGF-β1-mediated PI3K and MAPK pathways. Oncol. Rep. 41, 2975–2986. 10.3892/or.2019.7062 30896841

[B46] Zhang Y. Jiang C. Li X. Li K. (2020). Effect and safety of huangqi-guizhi-wuwu decoction and erxian decoction in the treatment of frozen shoulder: A protocol for systematic review and meta-analysis. Medicine 99, e20540. 10.1097/MD.0000000000020540 32502014 PMC7306347

[B47] Zheng H. Shen X. He Y. Yan X. Wang S. Yu A. (2020). Pharmacokinetic analysis of Huangqi Guizhi Wuwu Decoction on blood and brain tissue in rats with normal and cerebral ischemia-reperfusion injury by microdialysis with HPLC-MS/MS. Drug Des. devel. Ther. 14, 2877–2888. 10.2147/DDDT.S257020 PMC7382588 32764886

[B48] Zheng Y. Yang F. Han L. Gou X. Lian F. Liu W. (2019). Efficacy of Chinese herbal medicine in the treatment of moderate-severe painful diabetic peripheral neuropathy: A retrospective study. J. Diabetes Res. 2019, 4035861. 10.1155/2019/4035861 31950063 PMC6948321

