# Supplementary figures and images for: Integrated network pharmacology and serum metabolomics approach deciphers the anti-colon cancer mechanisms of Huangqi Guizhi Wuwu Decoction
Source: Front Pharmacol. 2022 Oct 13;13:1043252. doi: 10.3389/fphar.2022.1043252 (PMC9607907; doi:10.3389/fphar.2022.1043252)

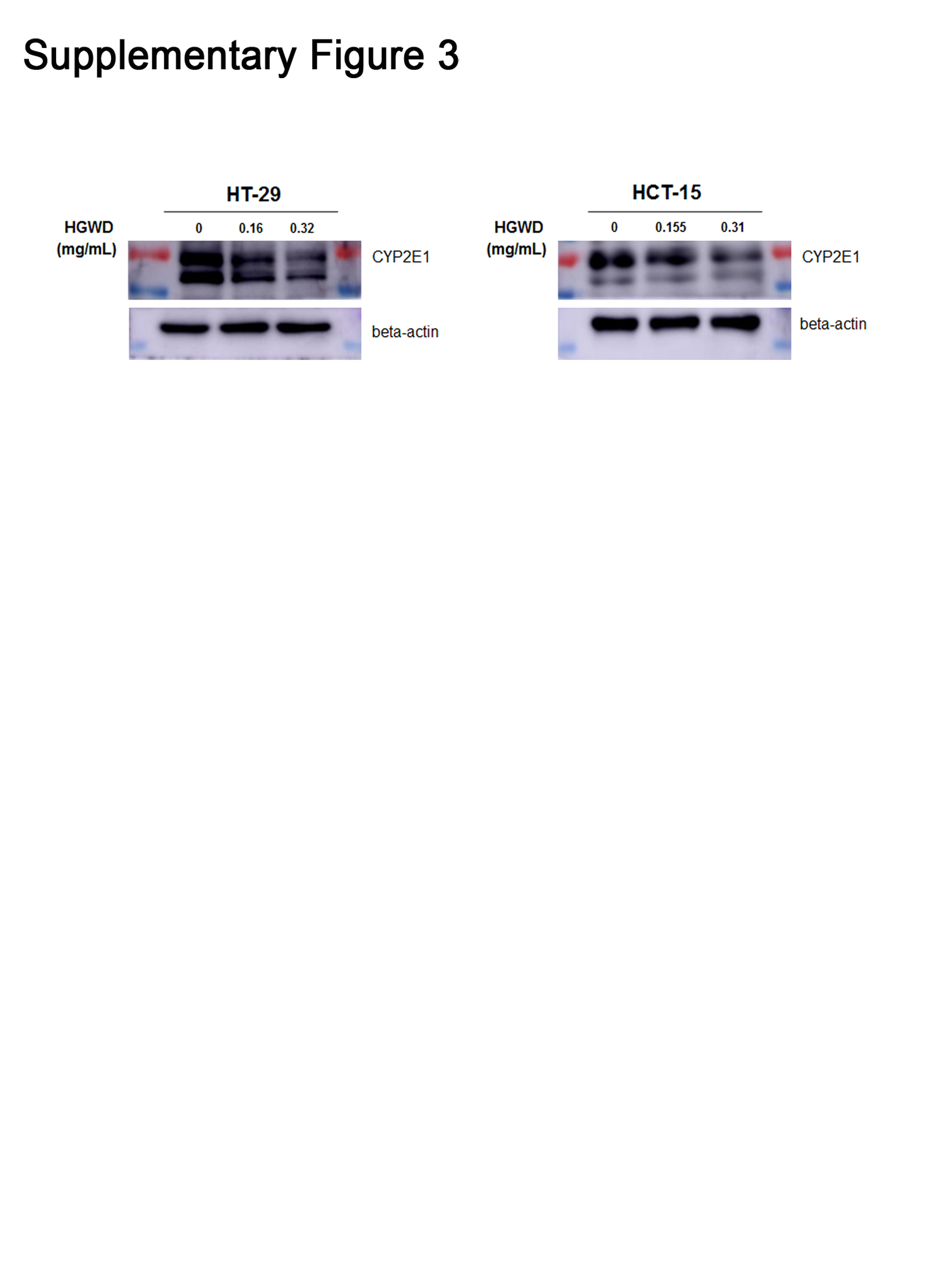

Supplement: Supplementary file 3 [file Image3.TIF]

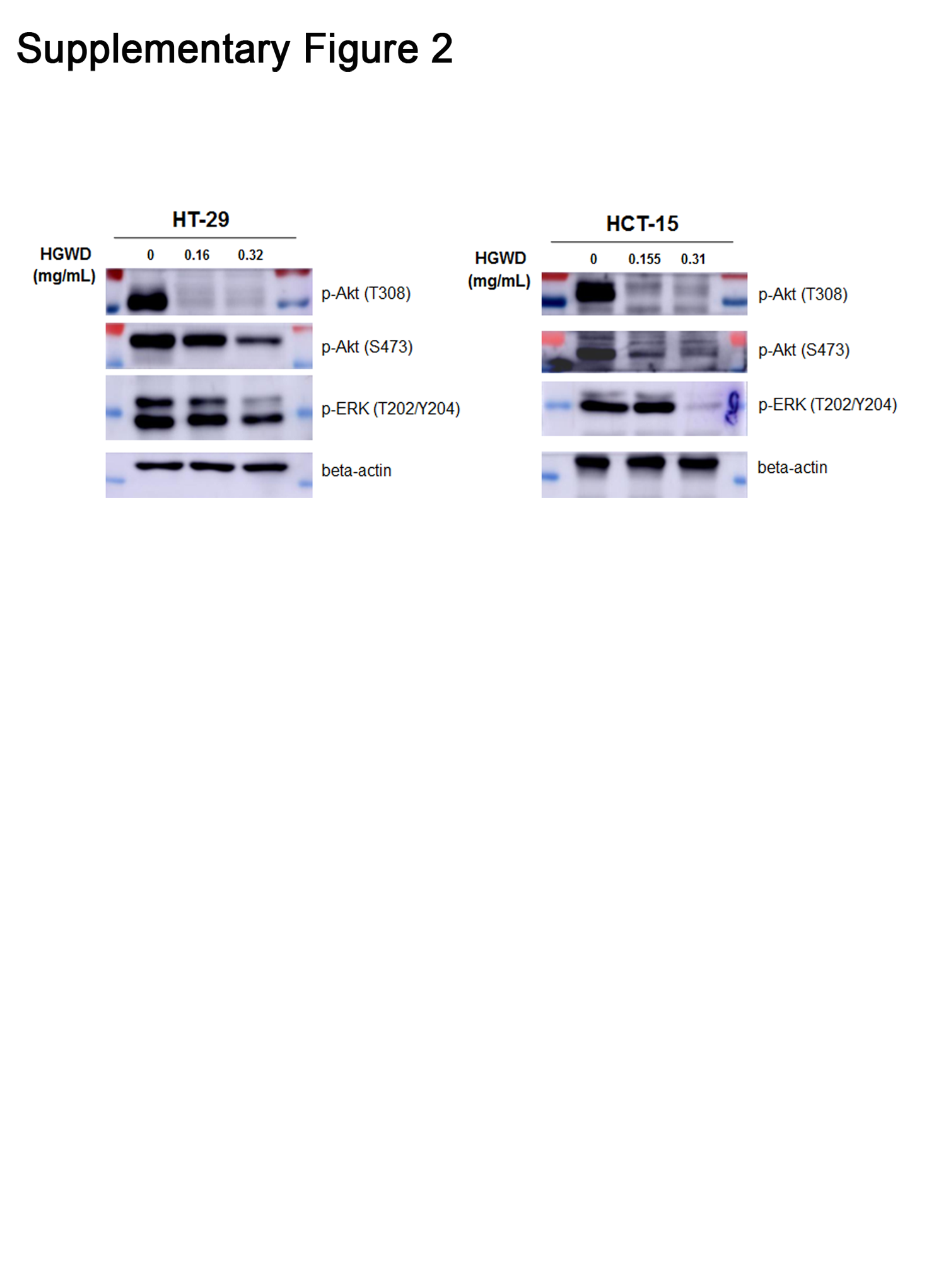

Supplement: Supplementary file 4 [file Image2.TIF]

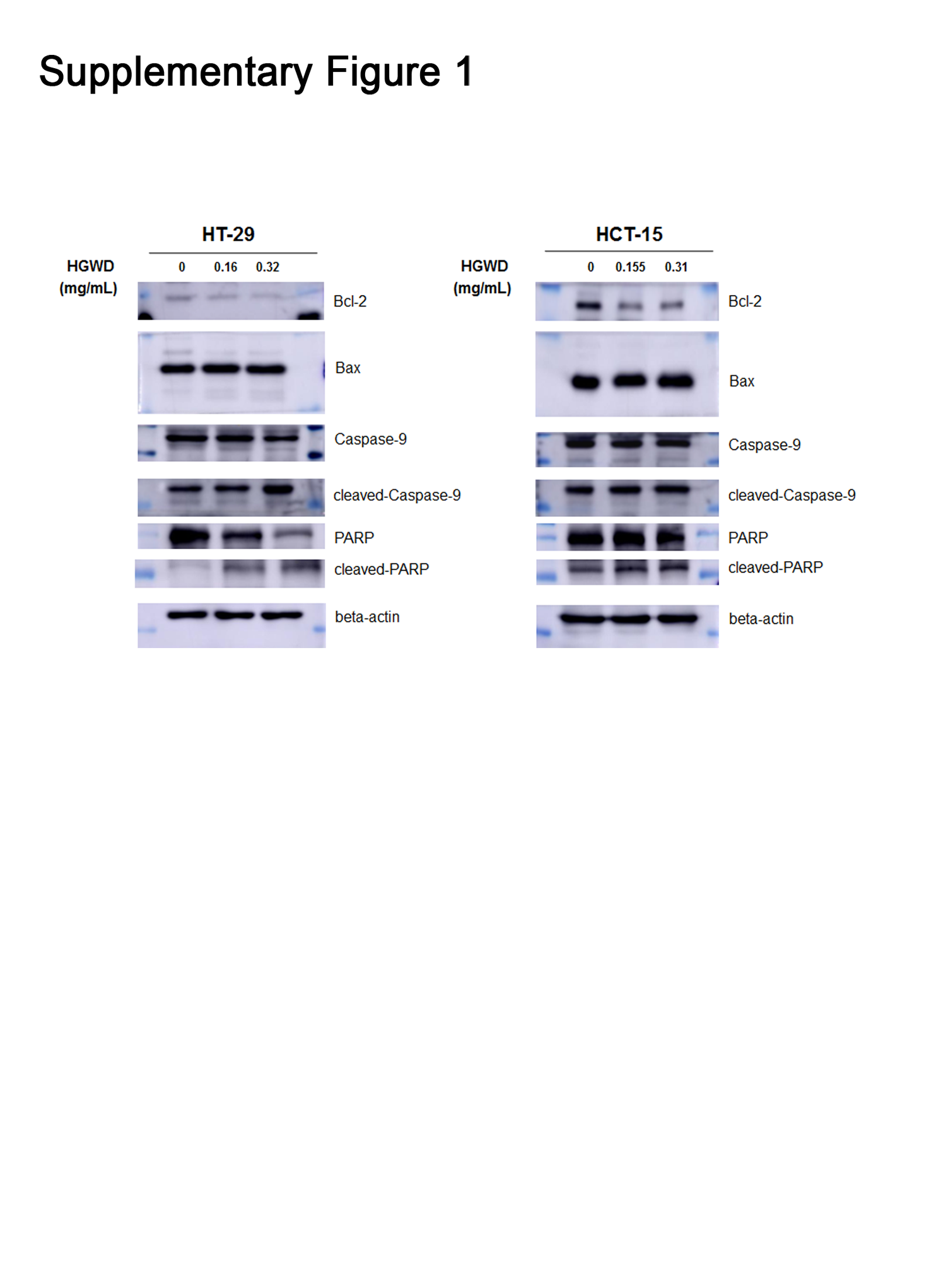

Supplement: Supplementary file 5 [file Image1.TIF]
